# Institutional Needs

**Published:** 1918-02-16

**Authors:** 


### Institutional Needs.

KEROCAIN (KERFOOT'S NOVOCAIN).

(Thos. Kerfoot and Co., Bardsley Vale, Lancs, and Bardsley House, London.)

Messrs. Kerfoot and Co. have been able to supply the profession, under licence from the British Board of Trade, with a product to which they have given the name Kerocain, and which is chemically, physiologically, and therapeutically identical with German Novocain. The increasing use made by surgeons of local anæsthesia renders such a product at the present time extremely welcome. It would be beside our present purpose to enter at any greater length into this interesting subject, especially as at least two text-books deal exhaustively with it. Of paramount interest, however, to us is that we have now produced in this country the substance which appears to be the ideal local analgesic. Kerocain has been thoroughly tested, and found to be in every respect reliable. It is sent out in various forms, tablets containing varying strengths of kerocain and adrenalin, with directions for their solution in varying quantities of distilled water so as to produce isotonic solutions of varying strengths of the active ingredients. We have also received a solution corresponding in strength to tablet A, ready for use, simply requiring sterilisation by being momentarily brought to the boiling point. We think Messrs. Kerfoot are to be congratulated upon their product.

